# Effects of the technique and drill design used during the osteotomy on the thermal and histological stimulation

**DOI:** 10.1038/s41598-020-77762-z

**Published:** 2020-11-26

**Authors:** Sergio Alexandre Gehrke, Tiago Luis Eliers Treichel, Jaime Aramburú Júnior, Piedad N. de Aza, Juan Carlos Prados-Frutos

**Affiliations:** 1 Department of Research, Biotecnos – Technology and Science, Cuareim 1483, 11100 Montevideo, Uruguay; 2 Department of Anatomy, Faculty of Veterinary, Universidade de Rio Verde, 104, Rio Verde, GO 75901-970 Brazil; 3 Department of Biotechnology, Universidad Católica de Murcia (UCAM), 30107 Murcia, Spain; 4 Instituto de Bioingenieria, Universidad Miguel Hernández, Avda. Ferrocarril s/n., 03202 Elche (Alicante), Spain; 5 Department of Medical Specialties and Public Health, IDIBO Group (High Performance Group in Research and Development of Biomaterials in Dentistry), Rey Juan Carlos University, Madrid, Spain

**Keywords:** Biotechnology, Cell biology, Medical research

## Abstract

The objective of our in vivo study was to compare the effects of the osteotomy on the thermal alterations, the bone healing and count of polymorphonuclear cells, comparing the drill design (cylindrical or conical) using continuous or intermittent movement. Twelve rabbits were used, which were made four osteotomies (n = 2 per tibia) to simulate the surgical drilling sequence for the installation of a dental implant at 8 mm of length and regular diameter. Four groups were proposed: group G1, cylindrical drill with continuous movement; group G2, cylindrical drill with intermittent movement; group G3, conical drill with continuous movement; and, group G4, conical drill with intermittent movement. Thermal mean variation was 6.91 ± 1.4 °C in group 1, 4.30 ± 1.3 °C in group 2, 2.78 ± 0.6 °C in group 3, and 2.77 ± 0.7 °C in group 4. Whereas the mean area of new bone formation was 1.00 ± 0.3 mm^2^ in group 1, 1.48 ± 0.3 mm^2^ in group 2, 2.20 ± 0.4 mm^2^ in group 3, and 2.43 ± 0.4 mm^2^in group 4. The mean count of polymorphonuclear cells, in the group 1 was 62.4 ± 5.9 cells, group 2 was 50.7 ± 4.2 cells, group 3 was 44.4 ± 3.7 cells, and group 4 was 42.4 ± 3.7 cells. The conical drill sequence produced a significantly smaller increase in temperature during both techniques (continuous and intermittent), more effective new bone formation and a smaller number of polymorphonuclear cells. During the osteotomy for the installation of implants, the professional must take to consider the drill design to perform a less traumatic surgical technique, which can improve and facilitate the healing of peri-implant tissues.

## Introduction

The success of dental implant therapy depends on the biological phenomenon of osseointegration in which there is a direct structural and functional connection between the living bone and the surface of an implant, sometimes a functional load^1^.

Numerous studies, mainly conducted on bovine models in vitro studies^2–9^, have concluded that one of the most determining factors in the osseointegration process is the heat produced in the bone perforation. It is recommended not to exceed a temperature higher than 47ºC to avoid bone necrosis and, as a result, the implant failure. The relationship between heat-generated osteotomy and bone perforation for dental implant placement is multifactorial, and its complexity has not been sufficiently studied^10,11^.

Alternative systems for performing osteotomy have been studied, such as piezoelectric devices, and laser systems (Nd: YAG, Er: YAG, Er, Cr: YSGG, CO_2_, and diode lasers) have been used for implant site preparation^1^. However, the factors involved in conventional systems such as drilling methods (single /sequential drilling, continuous/intermittent drilling, high speed / low speed, drill force/drill load, use a surgical guide), mode of irrigation (single/double irrigation, internal/external irrigation), the geometric characteristics of the drill or the implant site (compact or cancellous bone) have been described in greater detail^11^.

Thus, studies are comparing the drilling method: single or sequential drill (most frequently used).^3^ Others evaluate the types of continuous or intermittent movements performed during the osteotomy^5,8,12^. And others focus on the various irrigation techniques: internal, external, single or double^2,4,8,10–13^. Finally, one of the factors that have been studied the most is the one related to the geometry of the drill^6^. Most studies use helical drills^2,4,6^, followed by conical drills^2,4^, and less frequently, cylindrical drills^7^.

The common point of these studies is to look for the techniques, materials, and tools that produce the lowest temperature during osteotomy. It has been observed that strawberries that lack relief angles and have the smallest clearance between them increase bone temperature^6^. Likewise, the heat produced in the cortical bone and the most apical portion of the bone seems to be related to the drill geometry^7^. An appropriate irrigation technique could be crucial to avoid thermal damage to the bone in the areas of the highest friction with the drill^13^.

There is a lack of unanimity regarding the factors that are involved in the increase in temperature during osteotomy, and some of them, such as the use of cylindrical drills, have been little studied.

The objective of our study was to compare the effects of osteotomy on bone healing, relative to the impact of movement (intermittent or continuous) with two types of drill design (cylindrical or conical). Also, verify if there is a correlation between the temperature generated during the osteotomy, the amount of newly formed bone, and the number of polymorphonuclear (PMNs) cells after 30 days depending on the type of drill used and the type of technique.

## Materials and methods

Forty-eight osteotomies were prepared using two different drills design: drill sequence (2.0, 2.8 and 3.5 mm) to install a cylindrical titanium implant with 8-mm in length and 4.1-mm in diameter (Straumann, Basel, Switzerland) and, a conical drill sequence (2.0, 3.5 and 4.0 mm) to install a conical titanium implant with 8-mm in length and 4-mm in diameter (Implacil De Bortoli Ltda, São Paulo, Brazil). Four groups were proposed: group G1, cylindrical drill with continuous movement; group G2, cylindrical drill with intermittent movement; group G3, conical drill with continuous movement; and, group G4, conical drill with intermittent movement. The drilling speed applied for each model used followed the manufacturers' recommendations: Drill sequence for a cylindrical 4.1 mm implant for groups 1 and 2: drill diameters were 2.2 mm (used at 800 rpm), 2.8 mm (600 rpm), and 3.5 mm (500 rpm); All drill sequence for a conical 4.0 mm implant for groups 3 and 4 was 1500 rpm. In all groups the final drilling length was 8-mm. Groups G1 and G2 can be considered control groups, since cylindrical drill designs are the most traditionally used in osteotomies for the installation of implants and as control in several studies to evaluate the performance of new drill systems^3,5–8,13^.

**Figure 1:**
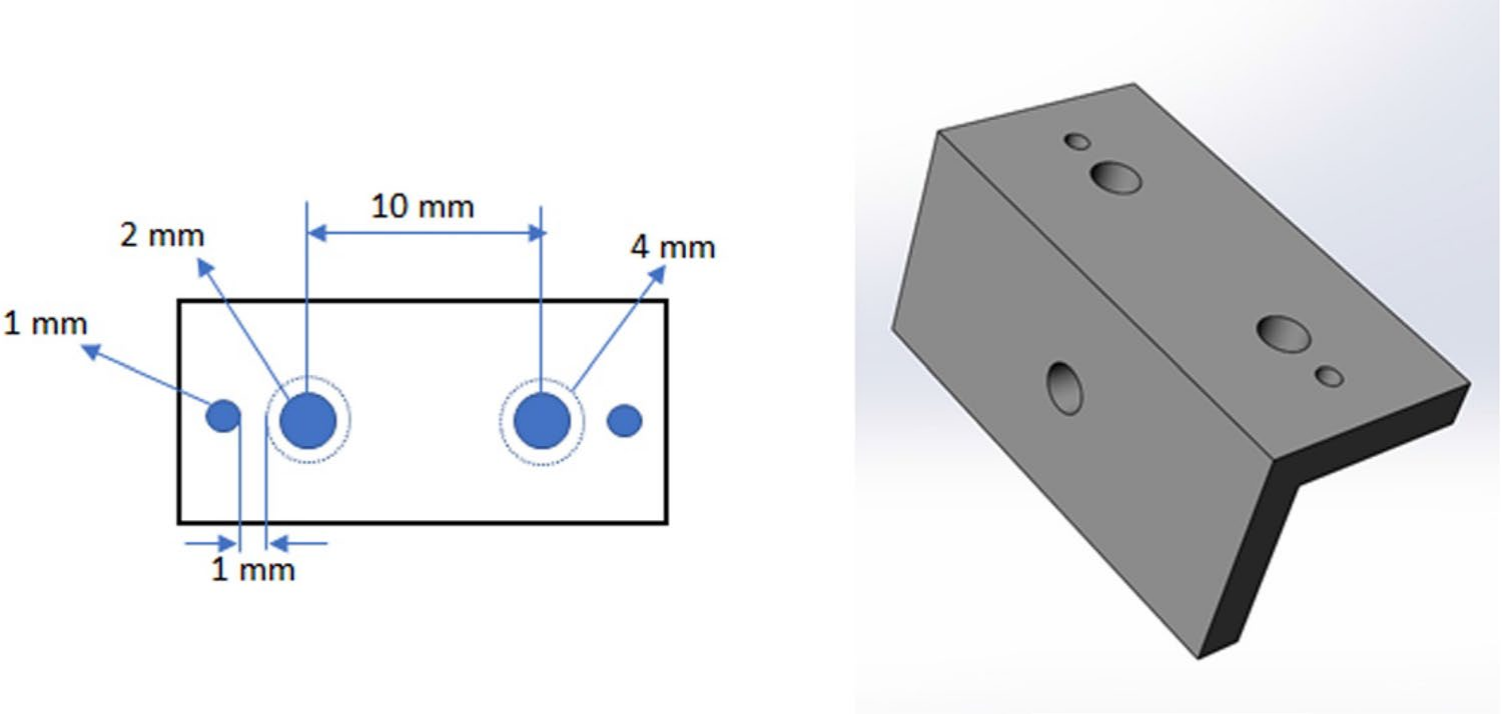
Demonstrative diagram of the fabricated guide for marking the osteotomy sites corresponding to each group (cylindrical or conical) and for positioning the type K sensors.

Twelve female white rabbits (New Zealand), with a weight of 4.0 ± 0.5 kg, were used. The animals received the standards care and management applied in the previous studies performed and described by our research group^14^. The international guidelines of the animal studies were applied. The study was approved by the Animal Experimentation Committee (Number 02-17UnRV), University of Rio Verde (Rio Verde, Brazil). Initially, a metallic guide (Fig. 1) was fabricated to mark the local of the osteotomies and the perforation to install the thermal sensors.

**Figure 2:**
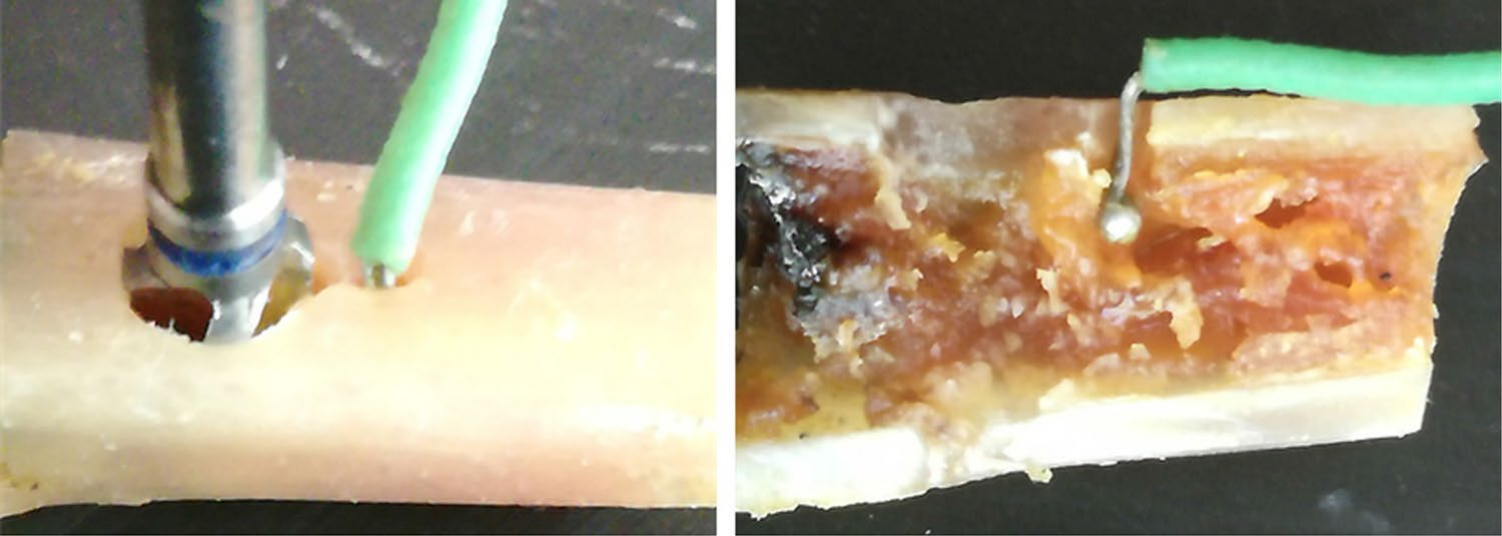
Schematic image of the osteotomy and the sensor positioned in a perforation localized 1 mm before and around to the final size of the osteotomy and 3 mm intrabone in each sample.

A type K thermocouple sensors were used to measure the maximum temperature reached during osteotomies, which is coupled to a portable digital thermometer model 1200 K (SalvTerm, São Paulo, Brazil) with a measurement range between − 50 and + 1300 °C and a resolution from 0.1 to 199.9 °C. Each sensor was positioned in a perforation localized 1 mm before and around to the final size of the osteotomy and 3 mm intrabone in each sample, in accordance with the representative image of Fig. 2, totaling two sensors per tibia. Then, the maximum temperature was measured during the osteotomy. After each measurement, the sensor was expected to return to initial temperature (at 33.5 ± 0.5 °C) and time interval of this event was recorded.

**Figure 3:**
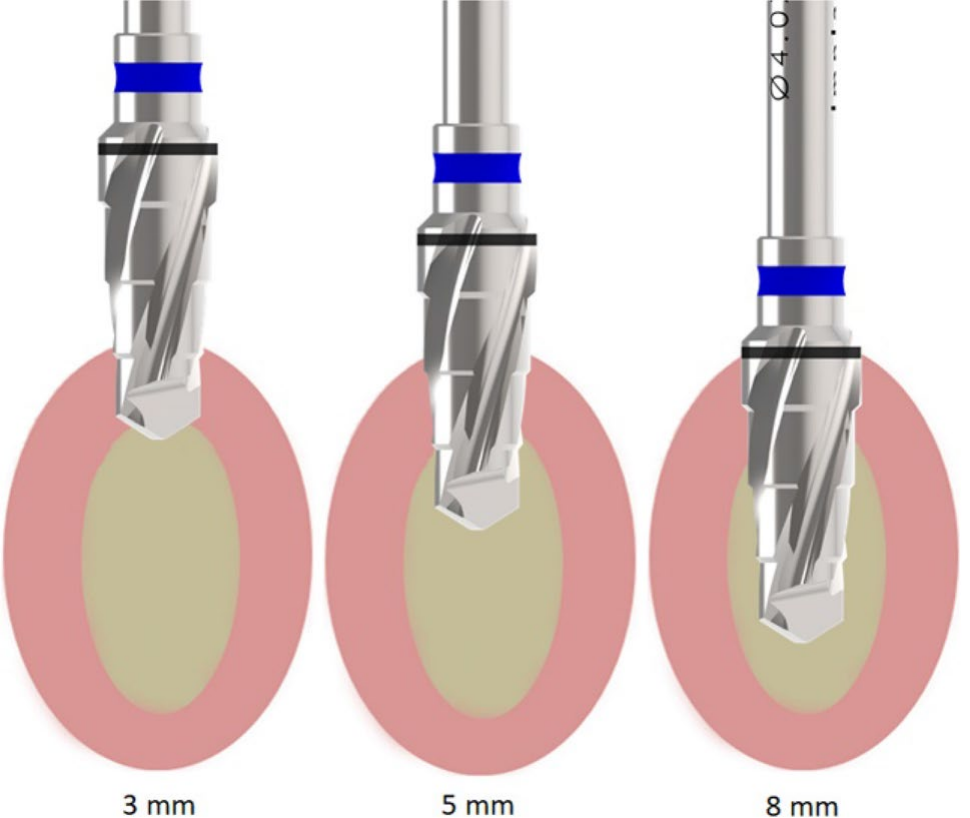
Schematic image of the drill insertion sequence during the osteotomy with movement.

A total of forty-eight osteotomies (*n* = 12 per group) were performed in both tibias (*n* = 2 per tibia). The osteotomy sites corresponding to each group were previously determined by lot, being was determined that in the right tibia would always be the osteotomies of group G1 in the proximal position and the osteotomies of group G3 in the distal position, and in the left tibia (osteotomies of group G4 in the proximal position and the osteotomies of group G2 in the distal position). For the intermittent movement, the drill sequence was inserted in the site using this protocol: 0–3 mm, 0–5 mm, and 0–8 mm (Fig. 3). Previous to realize the surgeries, the drills were marked to identify the 3 and 5 mm position.

**Figure 4:**
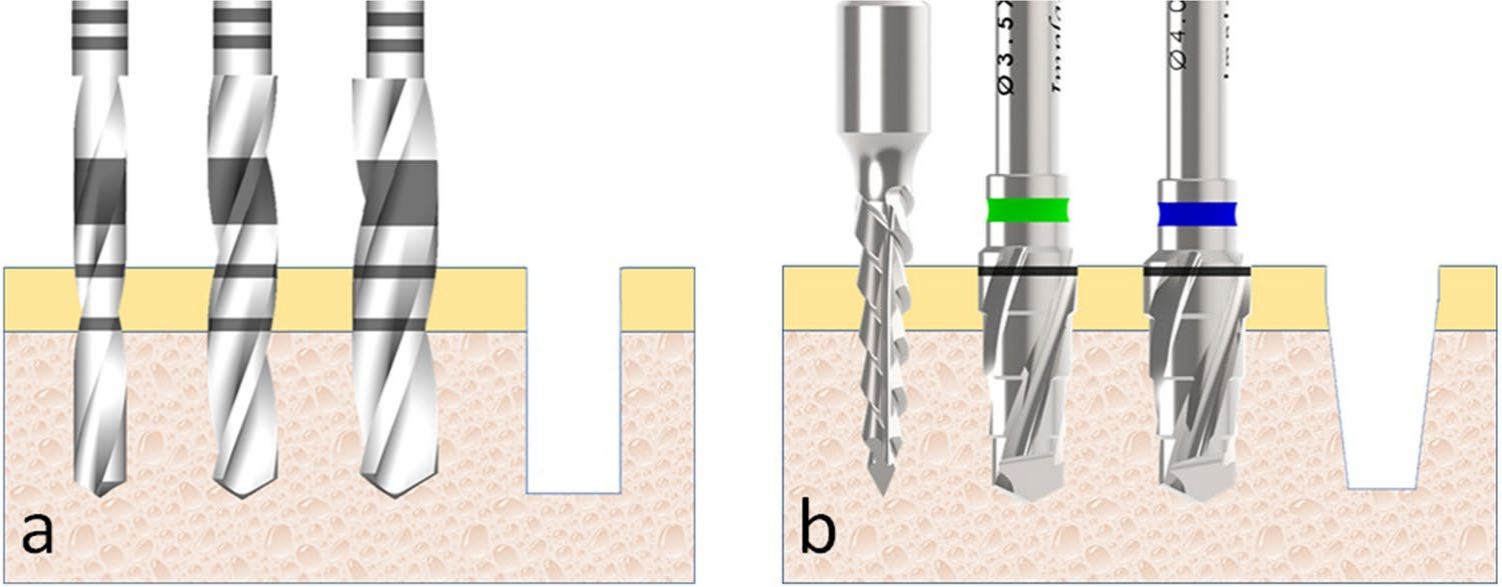
Representative schematic image of the drill sequence used for the osteotomy. (**a**) Osteotomy sequence for the G1 and G2 groups and (**b**) osteotomy sequence for the G3 and G4 groups.

Initially, the animals were anesthetized using a combination of 0.35 mg/kg of ketamine (Ketamine Agener; Agener União Ltda., São Paulo, Brazil) and 0.5 mg/kg of xylazine (Rompum Bayer S.A., São Paulo, Brazil), with the intramuscular application. Both tibias have scraped the hairs and cleansed with antiseptic solutions before the surgical procedures to avoid contamination. Then, an incision was performed initiating ~ 10 mm from the knee at the distal direction with a length at ~ 30 mm. The bone tissue was exposed, the metallic guide was positioned, and the local of the osteotomies were marked on the cortical bone using a carbide bur #699 (Meisinger, Neuss, Germany). Then, the sensors site was performed in a depth of 3 mm and, the osteotomies were performed using the drill sequence corresponding to each group (Fig. 4), under intense irrigation with saline solution at room temperature at 23 ± 1 °C.

Ten millimeters of the distance between the first osteotomy and the knee articulation was observed. Finally, a simple point suture was performed using an Ethicon nylon 4–0 (Johnson & Johnson Medical, New Brunswick, USA). After the surgeries, all medication was administered intramuscularly as follows: a single dose of 0.1 ml/kg of Benzetacil (Bayer, São Paulo, Brazil); three doses (one per day) of 3 mg/kg of ketoprofen (Ketoflex, Mundo Animal, São Paulo, Brazil). The euthanize was performed using an overdose of anesthesia at 30 days after the osteotomies and, the tibias were removed and immediately immersed in a 4% formaldehyde solution.

### Histomorphometric and histological analysis

Two weeks after immersion in the fixing solution, the samples were washed in running tap water per 12 h and gradually dehydrated in a progressive series of ethanol solution (60—100%). Then, the tibias were embedded in historesin (Technovit 7200 VLC, Kultzer & Co, Wehrheim, Germany), polymerized and cut in the central region of the osteotomies using a metallographic cutter machine (Isomet 1000; Buehler, Germany). After fixing the cuts on the slides, the samples were polished using a sequence of abrasive paper (180—1200 mesh) in a polishing machine (Polipan-U, Panambra Zwick, São Paulo, Brazil). The samples were stained using a picrosirus hematoxylin staining technique to evaluate the areas of new bone formation (nBF). Then, all biopsies were decolorized and new staining with hematoxylin and eosin (H&E) to assess the presence of polymorphonuclear cells in the entire osteotomies. Images using optical microscopy (Nykon E200, Tokyo, Japan) were captured of all samples.

**Figure 5:**
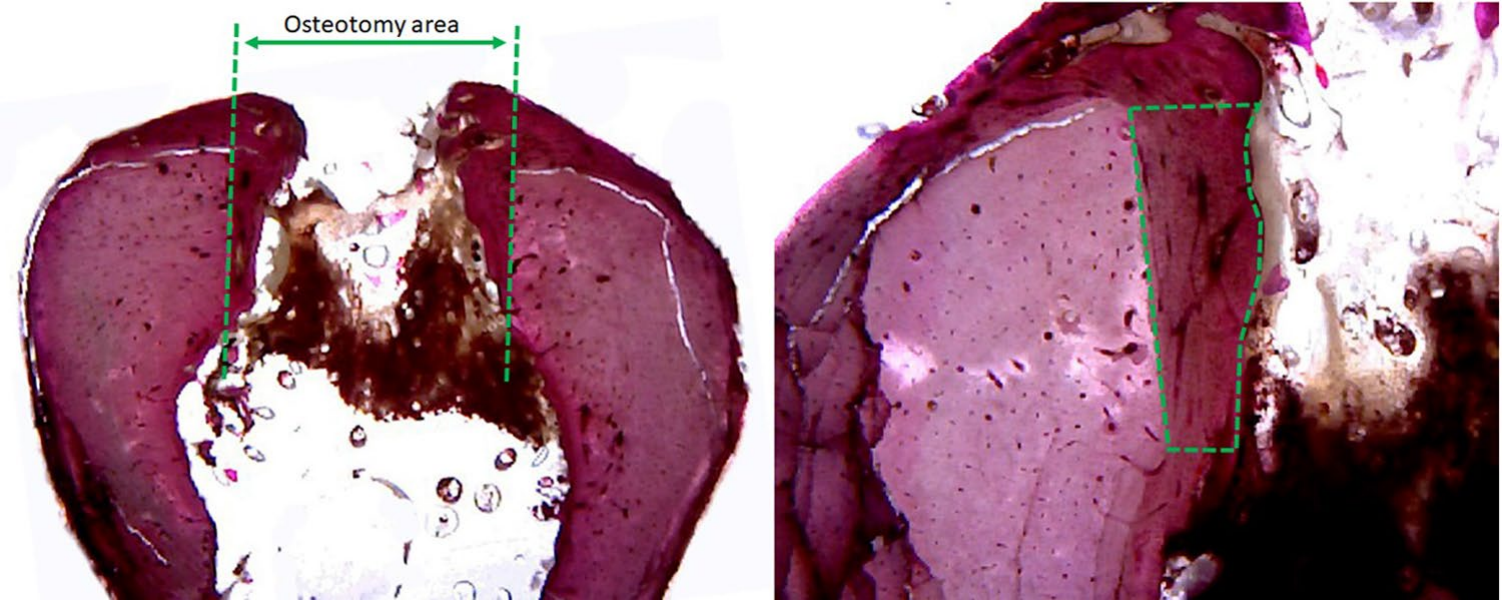
Representative image of the cortical area of new bone formation measured in all samples in both sides: (**a**) demarcation with the green line of the osteotomy and, (**b**) the measured area of the new bone.

The measurement of the area of nBF was performed considering the cortical portion in the 2 sides (right and left) where were made the cut by the drill in direction to the center of the perforation created, in accordance with the schematic image of Fig. 5. After the slides were stained, it was possible to see clearly, by the difference in color between the native bone tissue and the newly formed bone tissue. For each sample, an average was drawn up between the two sides measured.

**Figure 6:**
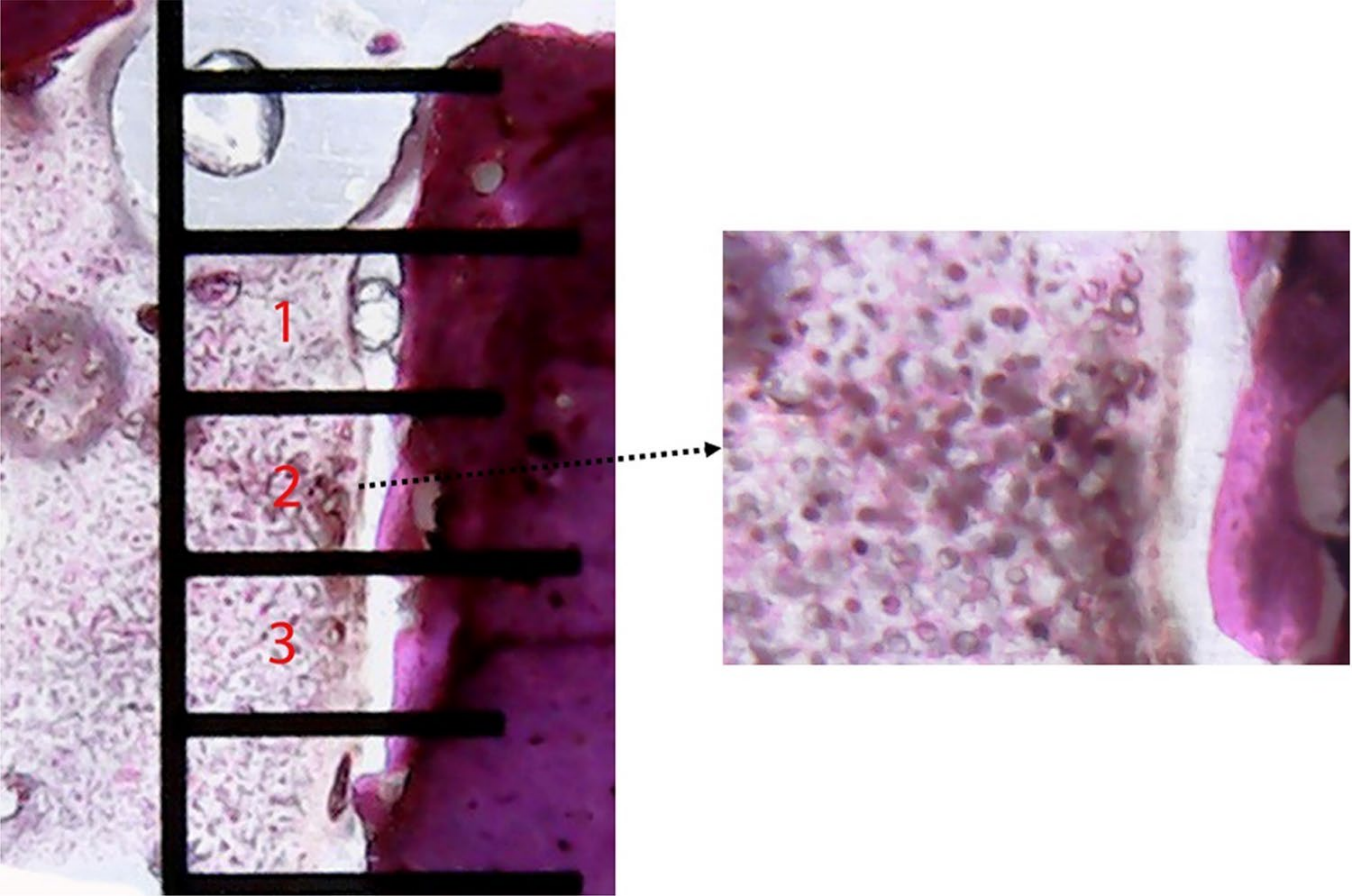
Representative image showing the 3 quadrangular sections measuring 500 × 500 µm used to count the inflammatory cells (ICs) number in all samples in both sides.

Inflammatory cells (ICs) number were obtained by counting all polymorphonuclear cells from six quadrangular sections measuring 500 × 500 µm (three on each side around the new bone formation area), in accordance with the schematic image of Fig. 6.

The area of nBF and the account of ICs were performed using the ImageJ program (National Institute of Health, Bethesda, USA).

### Statistical analysis

The ANOVA one-way statistical test was used followed by Bonferroni's multiple comparison test to determine the individual difference among groups. All analyzes were performed using GraphPad Prism version 5.01 for Windows (GraphPad Software, San Diego, California, USA). When *p* < 0.05 the differences were considered significant.

### Ethical approval

The present study was approved by the Animal Experimentation Committee (Number 02-17UnRV), University of Rio Verde (Rio Verde, Brazil). All applicable international, national, and/or institutional guidelines for the care and use of animals were followed.

### Informed consent

For this type of study, formal consent is not required.

## Results

During postoperative control, the sites where the surgeries were performed did not show signs of inflammation and/or infection, with healing free of problems or complications. All tibiae of all animals included in the present study could be recovered and used for due analysis.

**Figure 7:**
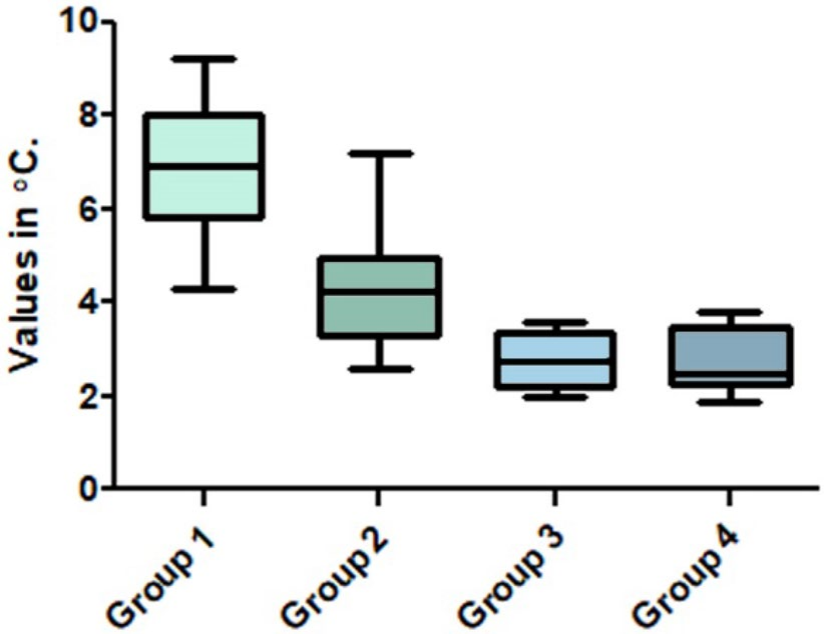
Box plots graph with the comparison of the temperature variation measured during the osteotomies in each group.

The temperature values measured during the osteotomies showed a difference from the initial temperature to the maximum temperature measured by passing the sequence of drills in each group in each condition, with the greatest variation being found in group G1 where cylindrical drills were used with continuous movement. While in groups G3 and G4, where conical drills were used, no significant differences were found regarding intermittent or continuous movement. The mean and standard deviation of maximum temperature registered in each group was: 40.4 ± 1.38 °C for group G1, 37.8 ± 1.62 °C for group G2, 36.3 ± 0.90 °C for group G3 and, 36.3 ± 0.80 °C for group G4. In general, the average waiting time for the temperature to return was 20 s after the end of drilling for groups G1 and G2 and 10 s for groups G3 and G4, which was considered a non-significant time. Then, the temperature variation values for each group and the statistical analysis of the data measured and compared between the groups are summarized in Table 1 and shown in the graph in Fig. 7.

**Table 1:** Bonferroni's multiple comparison test to compare the temperature variation values between the four groups with statistically significant difference.

Group Comparison	Means (in °C)	Mean of Diff	*p* value	95% CI
G1 vs G2	6.91 vs 4.30	2.608	0.0010*	1.435 to 3.781
G1 vs G3	6.91 vs 2.78	4.133	< 0.0001*	2.960 to 5.306
G1 vs G4	6.91 vs 2.77	4.142	< 0.0001*	2.969 to 5.315
G2 vs G3	4.30 vs 2.78	1.525	0.0015*	0.3519 to 2.698
G2 vs G4	4.30 vs 2.77	1.533	0.0283*	0.3602 to 2.706
G3 vs G4	2.78 vs 2.77	− 0.0083	0.9769	− 1.165 to 1.181

*Diff.* differences; *with difference statistical (*p* < 0.005), *CI* confidence interval; *°C* centigrade degree.

**Figure 8:**
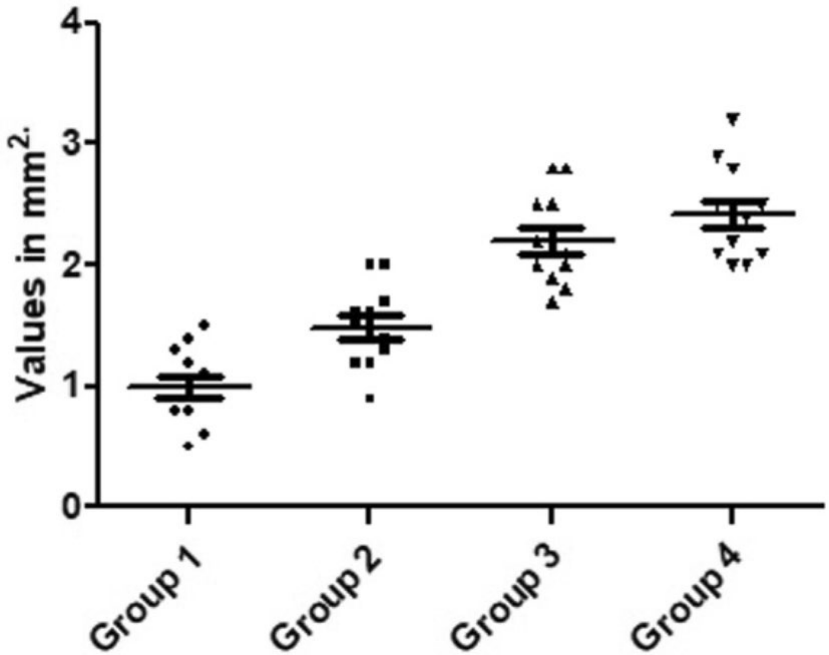
Graph with the distribution of the measured values comparing the new bone formation area for each group.

The measured values of the area of bone neoformation in the cortical portion of bone tissue were higher for groups 3 and 4 where osteotomies were performed with conical drills compared to groups 1 and 2 where osteotomies were performed with cylindrical drills. The values of the areas of bone neoformation measured as well as the statistical comparison between the groups are summarized in Table 2 and presented graphically in Fig. 8.

**Table 2:** Bonferroni's multiple comparison test to compare the measured areas of nBF values between the four groups with statistically significant difference with 30 days after the osteotomies.

Group Comparison	Means (in mm^2^)	Mean of diff	*p* value	95% CI
G1 vs G2	1.00 vs 1.48	− 0.4833	0.0029*	− 0.8755 to − 0.09117
G1 vs G3	1.00 vs 2.20	− 1.200	< 0.0001*	− 1.592 to − 0.8078
G1 vs G4	1.00 vs 2.43	− 1.425	< 0.0001*	− 1.817 to − 1.033
G2 vs G3	1.48 vs 2.20	− 0.7167	0.0003*	− 1.109 to − 0.3245
G2 vs G4	1.48 vs 2.43	− 0.9417	< 0.0001*	− 1.334 to − 0.5495
G3 vs G4	2.20 vs 2.43	− 0.2250	0.1626	− 0.6172 to 0.1672

*Diff.* differences; * with difference statistical (*p* < 0.005); *CI* confidence interval; *mm*^*2*^ square millimeters.

**Figure 9:**
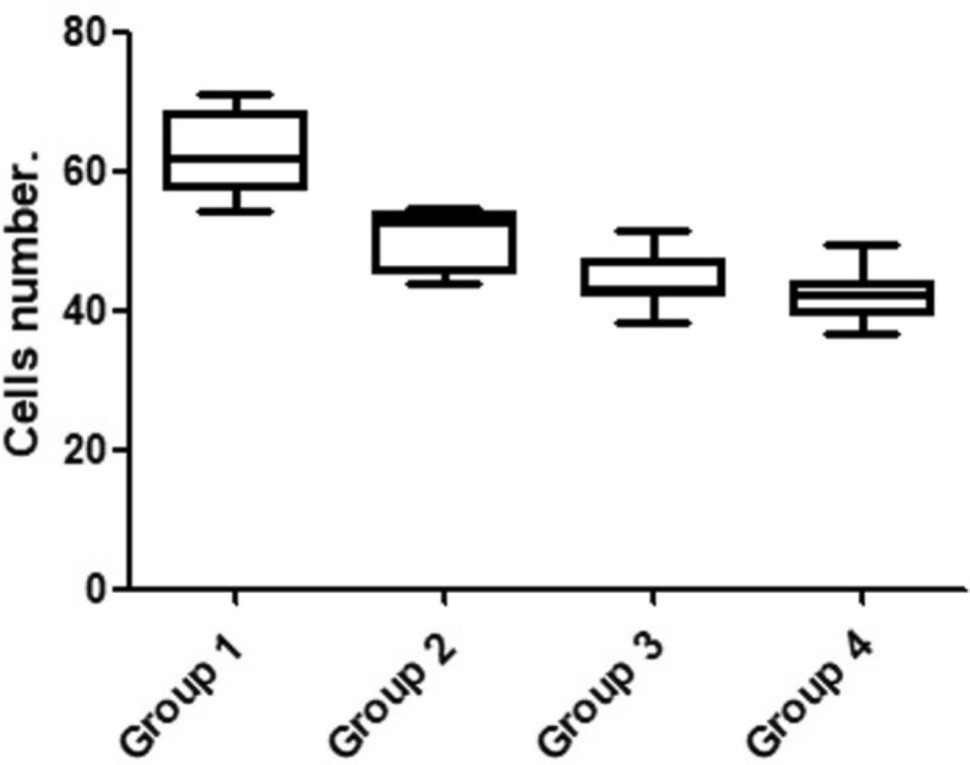
Graph with the distribution of the cells count for each group.

The polymorphonuclear cell count revealed, on average, lower values in the G4 group (42.4 ± 3.7 cells), followed by the G3 group (44.4 ± 3.7 cells), G2 group (50.7 ± 4.2 cells) and the highest value was found in the G1 group (62.4 ± 5.9 cells). The values of the inflammatory cells count as well as the statistical comparison between the groups are summarized in Table 3 and presented graphically in Fig. 9.

**Table 3:** Bonferroni's multiple comparison test to compare the ICs count between the four groups with statistically significant difference with 30 days after the osteotomies.

Group comparison	Means (cells)	Mean of diff	*p* value	95% CI
G1 vs G2	62.4 vs 50.7	11.68	< 0.0001*	6.627 to 16.72
G1 vs G3	62.4 vs 44.4	17.93	< 0.0001*	12.89 to 22.98
G1 vs G4	62.4 vs 42.4	20.00	< 0.0001*	14.95 to 25.05
G2 vs G3	50.7 vs 44.4	6.258	0.0011*	1.210 to 11.31
G2 vs G4	50.7 vs 42.4	8.325	0.0002*	3.277 to 13.37
G3 vs G4	44.4 vs 42.4	2.067	0.1744	− 2.981 to 7.115

*Diff.* differences; *with difference statistical (*p* < 0.005); *CI* confidence Interval.

The analysis of the statistical correlation between the temperature variation produced by the passing of the drill sequence of each group and the amount of bone neoformation at the osteotomy site after 30 days of healing showed different proportions according to the type of technique used (with intermittent movement or without). The temperature variation during the osteotomy and the newly formed bone produced by the osteotomy with cylindrical drills with continuous movement (group 1) and the amount in the area of ​​newly formed bone are not correlated, as shown by the very small multiple determination coefficients (*r* = 0.084), as well as with the use of cylindrical drills and intermittent movement (group 2), that showed a multiple determination coefficient of *r* =  − 0.029.

**Figure 10:**
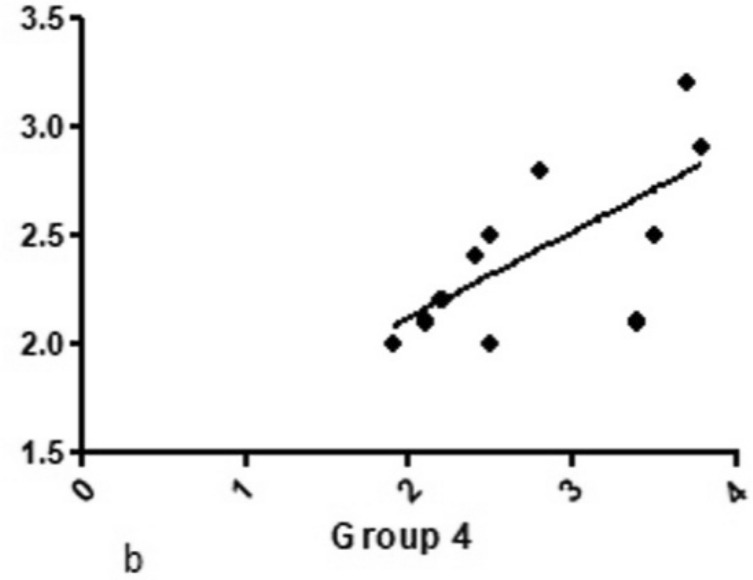
Graph of the positive correlation between the temperature variation and bone formation area for group 4 (conical drills with intermittent movement).

While in group 3, where a sequence of conical drills with continuous movement was used, although the multiple coefficients of determination were *r* = 0.300, which is not very high, it was much higher than groups 1 and 2. However, in group 4, where a sequence of conical drills with intermittent movement was used, the correlation was stronger, the multiple coefficients of the determination being *r* = 0.685 (Fig. 10).

**Figure 11:**
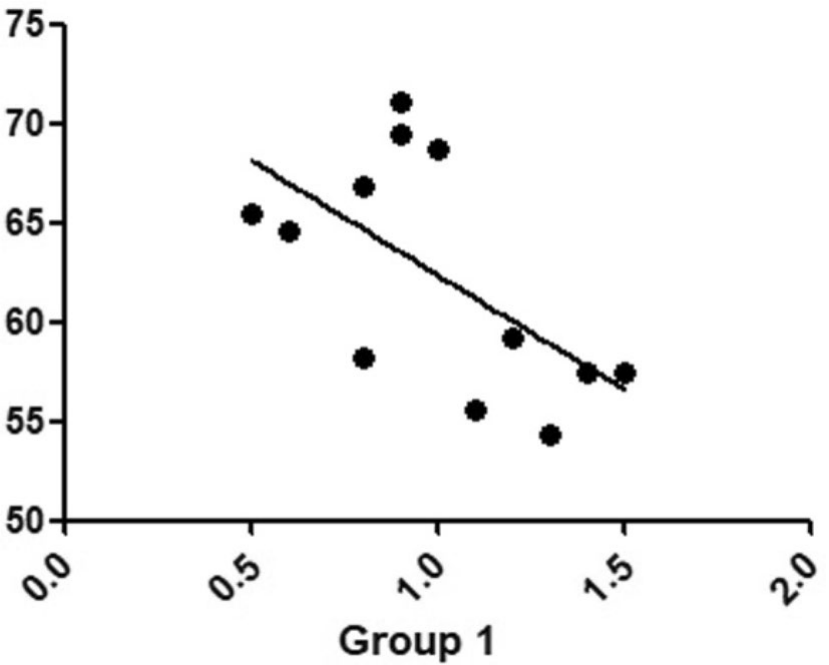
Correlation graph of the inflammatory cells number and bone formation area of the group 1.

When the correlation between temperature variation and the number of inflammatory cells was evaluated, all groups did not show a correlation between these two parameters tested. While in the correlation between the amount of bone neoformation at the osteotomy site after 30 days of healing and the amount of inflammatory cells counted, only in group 1 the correlation was stronger, the multiple coefficients of the determination being *r* = 0.6038 (Fig. 11), and in the other groups, no correlation was detected between these two parameters.

## Discussion

Most of the factors that influence the viability of the bone bed prepared for the insertion of the implants have been described in the literature and are the target of research with different bone models, such as synthetic bones (in vitro), bones of dead animals (ex vivo), live animals and humans (in vivo)^15,16^. The main differences between the ex vivo and in vivo experimental model are bone density and cellularity, which indicates cell viability^17^. However, factors related to the manipulation of tissues during surgical preparation for the installation of implants continue to be widely researched due to the emergence of new technologies and protocols. Our study sought to verify in vivo, using an animal model, the possible effects of heating caused during the osteotomy for the installation of an implant on the healing of bone tissue, and two different drill designs (cylindrical vs. conical) were tested and, two different milling techniques (continuous vs. intermittent movement). The results showed that depending on the drill design, the technique influences the results, that is, when using cylindrical drills, it is essential to use the intermittent movement technique, while when using conical drills, the technique does not interfere in the results. However, the use of conical drills generated less temperature variation and a greater area of neoformed bone in 30 days compared to the sites that were prepared with cylindrical drills.

Regarding the drills used in osteotomies for different implant models, due to a large number of implant shapes and systems on the market, the comparison between the different drill designs or formats and their relationship with bone temperature variation is impossible. However, some studies show that during osteotomy, regardless of the type of irrigation system used, the scaling of the drills used is suggested (start with drills of smaller diameter up to the planned diameter)^15,18–20^. In this sense, in the present work, two drill sequence models were selected that are available in most implant systems, that is, drills for the installation of cylindrical implants and drills for the installation of conical implants. Both models tested recommend the use of progressive augmentation during osteotomy using the same number of drills, in this case, 3 drills.

The use of intermittent movement, instead of continuous movement, is recommended by the literature, as it has advantages in temperature control, regardless of the type of irrigation system used. Studies that used intermittent movement showed that the temperature was more easily controlled and, in none of them, the temperature reached a critical level of 47 °C^6,15,19^. On the other hand, studies using continuous motion have exceeded the critical temperature in some cases^20–23^. However, most studies have not compared drill systems with different designs, as according to the results obtained in the present study, the use of intermittent movements is more necessary when cylindrical drills are used for the elaboration of osteotomies. This probably occurs because the tapered drills penetrate the bone site in a staggered manner due to their design, that is, differently from cylindrical drills, where the increase occurs by the diameter of each drill along the entire length of the osteotomy.

Since the discovery of osseointegration, the concepts of surgical technique and care for bone tissue have been improved. These advances aim to reduce surgical trauma and favor a better response of the tissues around the implant, and consequently, the achievement of osseointegration^24–26^. The excess heat generated during the osteotomy process can influence the repair process, causing hyperemia, necrosis, fibrosis, cell degeneration, and increased osteolytic activity, leading to failure of osseointegration^22,27^. In a recent study by our group using immunohistochemical analysis, measuring the peak NF-kB activation and its dissipation, it was demonstrated that the trauma generated during the drilling of bone tissue can alter the parameters of inflammation in these locations^28^.

The increase in bone temperature during bed preparation for implant installation is a multifactorial cause^17^. However, several precautions can prevent overheating and possible trauma to bone tissue, such as the use of intermittent drilling movements^20^, application of adequate force and rotational speed^18,21^, not using worn drills^6,15,20^, observation of bone tissue density^29^, decrease in drilling time^17^, and then use an appropriate irrigation technique^5,8^. Some authors used thermography to measure the temperature generated during osteotomies in their experiments^7,30^, while other authors used the technique of installing thermocouples, which are electrical devices with wide applications for temperature measurement^3,8,9^. In the present study, according to most studies, thermocouples were used to measure the heat generated in the cortical and bone marrow during osteotomies. Aghvami and collaborators considered the medullary cavity as a region of thermal dissipation, not showing a significant increase in temperature during drilling^31^. In addition, several authors described that bone density is much more important in raising the temperature than the depth of osteotomy^11,20,21^. Therefore, in this study, the thermocouples were inserted in a position with a depth of 3 mm.

Some factors used in our work, as they could influence the results, have been standardized. Thermocouples were inserted, at an average distance of 1.0 mm from the final edge of the drilling produced by the last drill in each tested system. This same distance between thermocouples and the drilling site was also adopted in other studies by our research group^3,8^. To control this distance, a guide was prepared, which was used to mark the exact location of the drilling with the first drill and the location of the thermocouple installation.

The cross-talk amongst inflammatory cells and cells related to bone healing is essential to the formation repair and remodeling of bone^32^, given that acute inflammation has been recognized as the first stage of fracture healing^33^. Inflammation is a crucial biological process for eradication of pathogens and maintenance of tissue homeostasis. Bone injury elicits an inflammatory response that is beneficial to healing when acute and highly regulated^34,35^. The polymorphonuclear cells provide an information of the inflammatory response of the organism to an injury. Neutrophils are phagocytic cells that engulf and destroy bacteria. The number of neutrophils is increased in infections, tissue necrosis, inflammatory diseases, metabolic disorders, and some leukemias. In addition to PMNs and monocytes/macrophages, the adaptive immune response has been implicated in the development of bone resorption^36,37^. Our results regarding the counting of PMNs cells in the areas where the osteotomies were performed showed a greater number of cells for the samples in group 1, compared with the other 3 groups. In addition, a positive correlation was observed between the lowest bone neoformation presented in the group 1 and the largest number of PMNs cells, which can be interpreted as an increase in trauma caused by the type of drill and the technique used for osteotomy.

Finally, testing the correlation between the type of drill used in osteotomies using different movement techniques and bone healing (area of newly formed bone), only the tapered drills showed a strong correlation when used with intermittent movements. As limitations of this study, we can report the bone model used (rabbit tibia) where the cortical bone is extremely dense, but the medullary portion is extremely soft. In addition, other variables must be tested, such as speed, strength, and time used for each type of drill. Although type K sensors have been used in several other studies published by our research group evaluating temperature control in bone surgery^3,8,38–40^, their sensitivity power can be reported as a limitations of the protocol used. New studies should be done with less time and more observation time and evaluate the potential for vascularization of these areas after osteotomies.

## Conclusions

Within the limitations of the present study, we can conclude that the use of conical drill systems for osteotomies produces a lower temperature variation and, consequently, improves the healing of bone tissue and showed a smaller number of polymorphonuclear cells in the areas undergoing healing. When cylindrical drill systems are used, it is essential to use a technique with intermittent movements, because in addition to decreasing the temperature variation during osteotomy, it improves the healing of bone tissue.
